# Measuring change in knowledge acquisition of Rwandan residents: using the American Board of Pediatrics International In-Training Examination (I-ITE) as an independent tool to monitor individual and departmental improvements during the Human Resources for Health program: an observational study

**DOI:** 10.1186/s12909-019-1617-8

**Published:** 2019-06-17

**Authors:** Natalie McCall, Christian Umuhoza, Cliff O’Callahan, Tanya Rogo, Diane Stafford, Aimable Kanyamuhunga, Peter T. Cartledge

**Affiliations:** 1 Yale University Rwanda Human Resources for Health Program, Department of Paediatrics, Centre Hospitalier Universitaire de Kigali (CHUK), PO Box 655, Kigali, Rwanda; 2 Department of Paediatrics, College of Medicine and Health Sciences, University of Rwanda, Kigali, Rwanda; 3 Middlesex Health, 90 S Main St, Middletown, CT 06457 USA; 4 Icahn School of Medicine at Mount Sinai, One Gustave L. Levy Place, New York, NY 10029-6574 USA; 5 Lucile Packard Children’s Hospital, Stanford University, 291 Campus Drive, Li Ka Shing Building, Stanford, CA 94305-5101 USA

**Keywords:** In-training examination, Formative feedback, Education, medical, Global Health, Rwanda, Internship and residency

## Abstract

**Background:**

Rwanda is the only African country to use the pediatric International In-Training Examination (I-ITE). The objectives of this study were to use the scores from the I-ITE to outline the baseline level of knowledge of Rwandan residents entering the pediatric residency and the trends in knowledge acquisition from 2012 to 2018, during the Human Resources for Health (HRH) Program, an education partnership between the Rwanda Ministry of Health and a consortium of US universities.

**Methods:**

A retrospective descriptive analysis of the I-ITE exam scores, taken by all Rwandan pediatric residents for five of the six academic years of the study period. Individual resident scores were weighted using the non-Rwandan I-ITE sites to minimise confounding from annual variations in exam difficulty. Statistical analysis included descriptives with ANOVA to compare variation in annual mean scores.

**Results:**

Eighty-four residents took 213 I-ITE exam sittings over the five exam cycles. The mean weighted I-ITE score of all residents increased from 34% in 2013 to 49% (*p* < 0.001) in 2018. The 32-point gap between the mean US-ITE and Rwandan I-ITE score in 2012–2013 was reduced to a 16-point gap in 2017–2018. First year resident (PG1) scores, which likely reflect the knowledge level of undergraduate medical students entering the residency program, increased from 34.8 to 44.3% (*p* = 0.002) between 2013 and 2018.

**Conclusions:**

The I-ITE is an independent, robust tool, measuring both learners and the institutional factors supporting residents. This is the first study to demonstrate that the I-ITE can be used to monitor resident knowledge acquisition in resource-limited settings, where assessment of resident knowledge can be a major challenge facing the academic medicine community. The significant increase in I-ITE scores between 2012 and 18 reflects the substantial curricular reorganisation accomplished through collaboration between Rwandan and US embedded faculty and supports the theory that programs such as HRH are highly effective at improving the quality of residency programs and undergraduate medical education.

## Background

Twenty-three African countries offered paediatric postgraduate training programs in 2013 [1]. Paediatric residents throughout the world are trained to gain and acquire skills, attitudes, and clinical judgment [2]; knowledge acquisition is also essential, and assessment using certified exams is an important tool in assessing this latter competency [3].

In the United States (US) monitoring this knowledge acquisition among pediatric residents has been accomplished over the last four decades through the use of an annual In-Training Examination (ITE) as a formative self-assessment instrument [4]. Although participation in the US-ITE is voluntary, program participation has increased from 55% in 2003 to 89% in 2015 demonstrating its formative value to US-residents and their programs [5, 6]. The objective of the ITE is to assess general pediatric knowledge, to track progress year-to-year and to compare scores with national peers. Research has shown that the pediatric ITE is a valid and reliable estimate of resident knowledge and can predict final performance in the General Pediatric Certifying Board examination [7].

The *International* In-Training Examination (I-ITE) is offered annually by the American Board of Pediatrics (ABP) to training institutions outside of North America. The I-ITE is a subset of 200 multiple-choice questions (MCQs) developed by board-certified subject matter experts [7]. ABP psychometric and test development experts match the multiple-choice questions (MCQs) to the current General Pediatrics Content Outline, and one or more medical editors review the I-ITE to ensure current/relevant information and to remove any Americanisms before each year’s administration. The I-ITE is constructed from ITE questions given previously to all US residents. Therefore the US comparison data is for US residents who took that specific, previous ITE. If questions are removed from the I-ITE, the US-ITE score is recalibrated with those items removed. The I-ITE was piloted with one training program in Lebanon in 2008, and made available to all countries in 2009. Ten countries used the I-ITE during our study period, namely: Bahamas, Italy, Kuwait, Lebanon, Oman, Qatar, Saudi Arabia, Singapore and the United Arab Emirates (UAE).

Rwanda is a small country in East Africa with a population of 10.5 million people according to the last census in 2012, a GDP of 787 USD per capita in 2018 [8]. Health expenditure per capita is 57 USD, which less than 1% of US health expenditure of 9536 per capita [9]. There is a significant shortage of healthcare workers, with only 15 paediatricians in the public sector in 2012, representing one Paediatrician per 700,000 population [10]. The Rwandan residency program at the University of Rwanda was strengthened by the technical partnership with the Human Resources for Health (HRH) Program, launched in August 2012 as a $150million US Government and Global Fund-funded initiative to address the lack of qualified health professionals in Rwanda. The long-term goal of the HRH Program is to build the health education infrastructure and health workforce needed to create high quality sustainable healthcare in Rwanda. In this program, an unprecedented consortium of over 20 respected US Universities partnered with the Government of Rwanda and deployed US-trained faculty with advanced clinical training and experience [11–13]. The HRH program supports faculty in the medical and surgical specialities, nursing, midwifery public health, and hospital management [14–16]. A recent systematic review of strategies to improve health-care provider practices in low-income and middle-income countries (LMICs) demonstrated that training or supervision programs and group-problem solving programs were the most likely to see improvements in healthcare provider practices [17]. The HRH program models these strategies.

Within paediatrics, the US institution faculty making up the HRH team has included general paediatricians and paediatric subspecialists who have worked alongside local University of Rwanda (UR) academic and Ministry of Health clinical staff to develop and support the educational, research and administrative activities of the Department of Paediatrics [11, 13]. During the period of the study, 84 residents undertook one or more I-ITEs. Forty-four graduated with a further 34 HRH-trained residents expected to graduate in the future, which will increase local paediatric workforce by close to 500% and hopefully make a difference to the community.

## Methods

### Aims

This study aimed to use a completely independent high-quality tool, the I-ITE, to measure the baseline level of paediatric knowledge of new residents entering the paediatric residency, and the trends in knowledge acquisition by Rwandan paediatric residents, from 2012 to 2018.

### Study design and site

We performed a retrospective analysis of I-ITE scores of all Postgraduate (PG) paediatric residents enrolled from 2012 to 2018 in the University of Rwanda (UR), the only university in Rwanda with a paediatric residency program. Our reporting of this observational study is in accordance with the Strengthening the Reporting of Observational Studies in Epidemiology (STROBE) checklist [18].

### Implementation of I-ITE in Rwanda

No Rwandan resident had previously taken the I-ITE before the HRH Program. We contracted with the American Board of Pediatrics to utilise the I-ITE in Rwanda for five of the six academic years of the study period, namely: November 2012, March 2014, April 2016, March 2017 and May 2018. However, we were unable to administer the I-ITE in 2015 due to financial and organisational barriers.

### Participants

All Postgraduate (PG) paediatric residents enrolled in the program from 2012 to 2018 sat the I-ITE.

### Data collection and management

Following standard ABP protocol, we administered the best-of-five MCQ I-ITE electronically using password protected computers connected to the internet and proctored by faculty. The ABP provided us with summary data for the US ITE and non-Rwandan International-ITE scores in electronic format along with raw scores for all the Rwandan PG residents, and data was password protected in an Excel spreadsheet.

### Sample size calculation

No sample size calculation was undertaken as all residents in the program sat the I-ITE throughout the study period, and we analysed all results.

### Weighting annual scores

We weighted exam scores for variation in difficulty. Although the difficulty of the questions in the I-ITE are relatively stable, there is still statistically significant variation year-to-year [19]. The ABP provided summary data in the form of counts, means and standard deviations for the US-ITE and non-Rwandan I-ITE for each year. We did not include an analysis of the raw US and non-Rwandan I-ITE sites but rather undertook a post-hoc analysis of the summary data of the US-ITE and non-Rwandan I-ITE scores. This analysis demonstrated that there was a significant difference in the difficulty year-to-year (ANOVA, *p* < 0.0001, Table 1). Therefore, to minimise confounding from this annual variation in exam difficulty, we weighted individual resident scores using the results from other non-Rwandan I-ITE sites. We calculated an annual weighting factor by dividing the mean non-Rwandan I-ITE score for each year by the mean of the non-Rwandan I-ITE scores for all five exam sittings of the study period (Table 1). Each resident score was then weighted by dividing their I-ITE score by the annual weighting factor. We chose to use the non-Rwandan I-ITE scores to calculate the weighting (rather than the larger US cohorts) as other International-ITE training sites would have been more likely to encounter similar challenges (e.g., language, pathology relevance, etc.). Each resident examinee I-ITE score was also converted into a z-score based on the mean and standard deviation of the non-Rwandan I-ITE scores for that year.

**Table 1:** Weighting of annual mean I-ITE scores based on other International exam centers and Annual Mean Weighted Rwandan I-ITE scores

		2012–13	2013–14	2015–16	2016–17	2017–18	*p*-value
	International Countries	Italy	Bahamas	Italy	Bahamas	Bahamas	Not applicable
		Kuwait	Italy	Kuwait	Italy	Italy	
		Lebanon	Kuwait	Lebanon	Kuwait	Kuwait	
		Oman	Lebanon	Oman	Lebanon	Lebanon	
		Qatar	Oman	Qatar	Qatar	Qatar	
		Singapore	Qatar	Singapore	Singapore	Saudi Arabia	
		UAE	Singapore	UAE	UAE		
			UAE				
All residents	US scores	66.0 (±10) (*n* = 9138)	62.0 (±10) (*n* = 9300)	66.0 (±10) (*n* = 9138)	60.2 (±8) (*n* = 10,965)	65.0 (±8) (*n* = 11,014)	*p* < 0.001^b^
	Non-Rwandan International scores	60.8 (±11) (*n* = 1024)	59.8 (±10) (*n* = 1128)	64.8 (±11) (*n* = 823)	56.9 (±10) (*n* = 502)	62.1 (±11) (*n* = 704)	*p* < 0.001^b^
	Annual weighting factor	0.998	0.983	1.064	0.935	1.020	–
	Rwandan non-weighted (raw) scorses	34.3 (±7.5) (*n* = 27)	38.8 (±8.6) (*n* = 41)	47.1 (±9.0) (*n* = 50)	43.5 (±7.0) (*n* = 49)	49.5 (±6.2) (*n* = 46)	*p* < 0.001^a^
	Rwandan weighted scores	34.3 (±7.5) (*n* = 27)	39.5 (±8.7) (*n* = 41)	44.3 (±8.4) (*n* = 50)	46.5 (±7.4) (*n* = 49)	48.6 (±6.1) (*n* = 46)	*p* < 0.001^a^
	Rwandan mean z-score	−2.57 (CI: − 2.85 to − 2.28) (*n* = 27)	−2.18 (CI: − 2.46 to − 1.89) (*n* = 41)	−1.78 (CI: − 2.04 to − 1.52) (*n* = 50)	−1.38 (CI: − 1.59 to − 1.17) (*n* = 49)	−1.30 (CI: − 1.50 to − 1.11) (*n* = 46)	*p* < 0.001^a^
PG1 residents	US PG1 scores	59 (±8) (*n* = 2993)	54 (±8) (*n* = 3062)	59 (±8) (*n* = 2993)	55 (±7) (*n* = 3545)	61 (±8) (*n* = 3595)	*p* < 0.001^b^
	Non-Rwandan International PG1 scores	57 (±10) (*n* = 286)	55 (±10) (*n* = 299)	59 (±10) (*n* = 214)	52 (±10) (*n* = 127)	55 (±10) (*n* = 167)	*p* < 0.001^b^
	Rwandan weighted PG1 scores	34.8 (±8.4) (*n* = 16)	35.1 (±7.2) (*n* = 14)	40.6 (±4.9) (*n* = 15)	43.7 (±7.9) (*n* = 12)	44.3 (±6.7) (*n* = 7)	*p* = 0.002^a^
	Rwandan PG1 mean z-scores	−2.15 (CI: −2.59 to −1.73) (*n* = 16)	− 2.00 (CI: − 2.39 to − 1.60) (*n* = 14)	−1.60 (CI: − 1.90 to − 1.30) (*n* = 15)	−1.16 (CI: − 1.64 to −0.67) (*n* = 12)	−0.98 (CI: − 1.60 to − 0.36) (*n* = 7)	*p* < 0.001^a^

*Abbreviations*: *CI* 95% Confidence interval

^a^ANOVA of total mean scores for PG level was undertaken

^b^Post-hoc ANOVA ± Standard Deviation

### Statistical analysis

We used the Statistical Package for the Social Sciences (SPSS) (IBM SPSS Statistics for Windows, Version 24, Armonk, NY: IBM Corp). In order to analyse increases in weighted scores and z-scores during the five sittings, we conducted an ANOVA and used a repeated-measures ANOVA to analyse if there was an increase in scores within each class.

### Ethical considerations

We paid particular attention to potential ethical issues and resident confidentiality. Faculty discussed the rationale and context for the exam before providing each resident with their score and ABP-provided subject breakdown. Residents’ results are otherwise only available to faculty and used for resident development and advising purposes**.** No incentives were offered, no potential risks were identified, and participation was deemed as consent. The Institutional review board (IRB) of the University of Rwanda (UR) approved the project proposal. Ref: 027/CMHS_IRB/2018.

## Results

### Participants

Eighty-four residents completed a total of 213 I-ITE sittings (Table 1) during the study period. Each resident sitting the I-ITE earned a score out of 100 (percentage), and scores are described as percentages throughout. No demographic data (age, gender, etc.) is available from the ABP.

### Annual variation and weighting

**Fig. 1:**
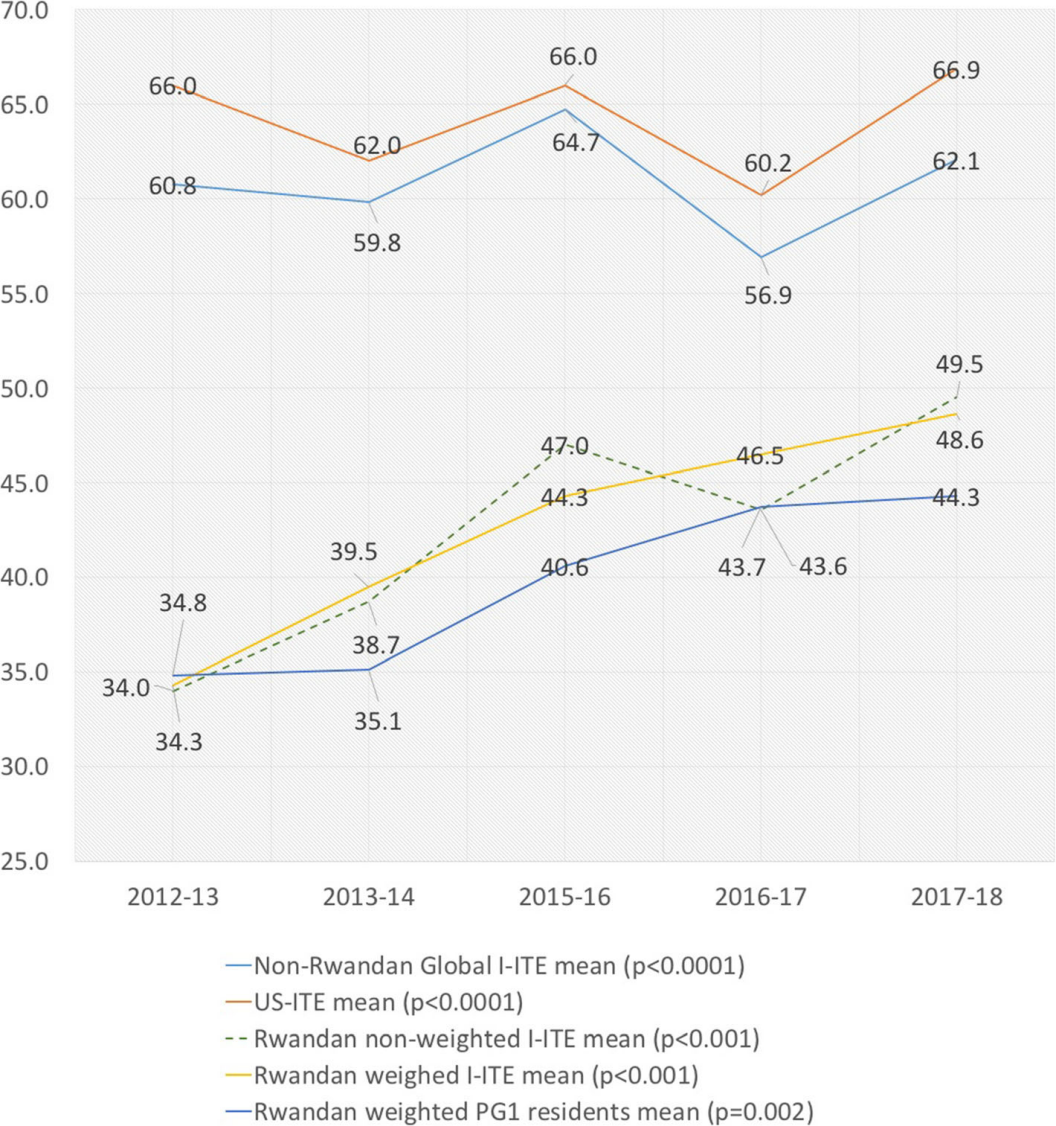
Annual variation in US-ITE, Non-Rwandan International-ITE and Rwandan Mean I-ITE scores. *p*-values represent ANOVA within category

We observed a significant fluctuation in the difficulty of the I-ITE in both the US-ITE and the International-ITE (Fig. 1) manifested a significant variation (*p* < 0.0001) in the mean scores from these sites. Therefore, we used the mean non-Rwandan I-ITE score to weight each Rwandan resident’s I-ITE score (Table 1).

### Changes in I-ITE scores

**Fig. 2:**
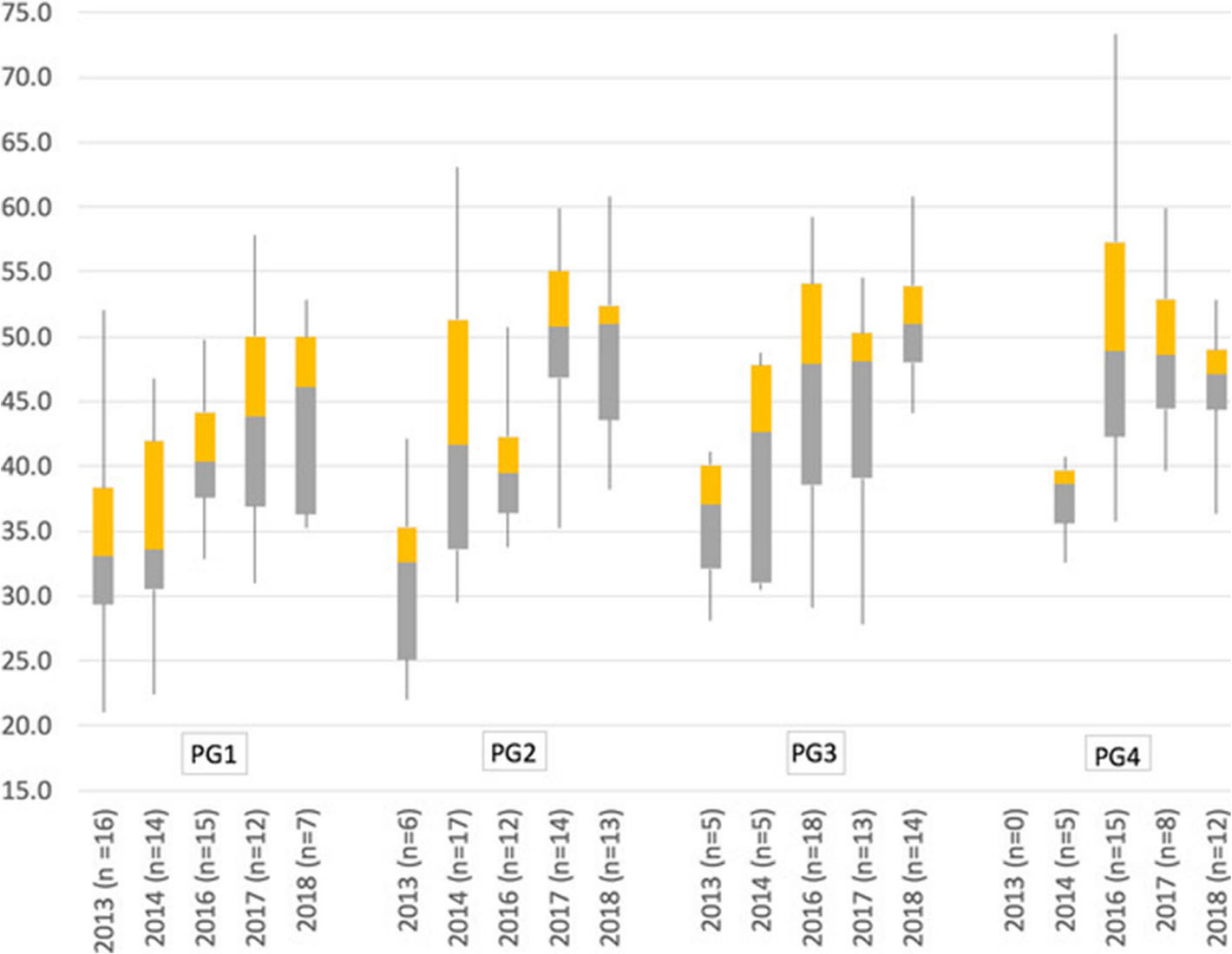
Box-plot of weighted Rwandan scores by level of PG training. Box-plot represents: Medians, interquartile ranges, min and max scores. *There were no PG4 residents in the program in 2012-13 due to no recruitment of residents

We demonstrate a significant increase in the performance of residents during the period of the HRH program with mean weighted-scores of all the residents increasing from 34.3 in 2013 to 48.6 (*p* < 0.001) in 2018 (Table 1). US-ITE scores were higher than the other non-Rwandan I-ITE scores (Fig. 1), but the 32-point gap between the mean US-ITE and Rwandan I-ITE score in 2012–2013 was reduced to a 16-point gap in 2017–2018. The pairwise increase was particularly significant between PG1 and PG2 and for the resident cohorts with more years of exposure to the HRH program during their training (Table 2 & Fig. 1). We surmise that PG1 scores are likely to reflect the knowledge level of undergraduate medical students (Table 1, Fig. 2) and demonstrate that the mean score of these entering residents increased from 34.8 in 2012 to 44.3 in 2018 (*p* = 0.002).

**Table 2:** Annual Mean Weighted Rwandan I-ITE scores by graduation class

Academic year of exam (year levels examined in this class)	2012–13	2013–14	2015–16	2016–17	2017–18	All years	Repeated measures ANOVA
Class of 2014 (PG3 & 4)	36.3 (±5.0) (*n* = 5)	37.9 (±3.1) (*n* = 5)	–	–	–	37.1 (±4.0) (*n* = 10)	*p* = 0.294
Class of 2015 (PG2 & 3)	31.4 (±6.9) (*n* = 6)	40.1 (±8.6) (*n* = 5)	–	–	–	35.3 (±8.6) (*n* = 11)	*p* = 0.012
Class of 2016 (PG1, 2 & 4)	34.8 (±8.4) (*n* = 16)	43.4 (±9.6) (*n* = 17)	49.9 (±9.8) (*n* = 15)	–	–	42.6 (±11.0) (*n* = 48)	*p* < 0.001
Class of 2017 (PG1, 3 & 4)	–	35.0 (±7.3) (*n* = 14)	46.3 (±9.7) (*n* = 8)	48.5 (±5.6) (*n* = 10)	–	42.0 (±9.7) (*n* = 32)	*p* < 0.001
Class of 2018 (PG2, 3 & 4)	–	–	40.6 (±4.9) (*n* = 12)	44.5 (±7.8) (*n* = 13)	46.6 (±4.5) (*n* = 12)	43.9 (±6.3) (*n* = 37)	*p* = 0.002
Class of 2019 (PG1,2 &3)	–	–	40.6 (±4.9) (*n* = 15)	49.5 (±7.1) (*n* = 14)	51.4 (±5.1) (*n* = 14)	47.0 (±7.4) (*n* = 43)	*p* < 0.001
Class of 2020 (PG1 &2)	–	–	–	43.7 (±8.0) (*n* = 12)	49.6 (±6.6) (*n* = 13)	46.7 (±7.8) (*n* = 25)	*p* < 0.001

± Standard Deviation

### Progression of year-groups

In order to analyse progression throughout time in the program, we created resident cohorts that were “tracked” by year of predicted graduation (Table 2). We observe a significant increase of the mean I-ITE score for the two I-ITE sittings taken by the class of 2014 (PG3/PG4 only) from 37.1 to 46.7 for the two I-ITE sittings taken by the class of 2020 (PG1/PG2 only), (Table 2, ANOVA, *p* < 0.001). We also demonstrate a greater progression in the increase of scores among residents who began residency in 2012 and later, while HRH was present.

## Discussion

Our objective was to use a globally available, independent monitoring tool of high quality that could objectively measure trends in knowledge acquisition by Rwandan paediatric residents from 2012 to 2018. With this study, we demonstrate a significant increase in I-ITE scores during this period, along with a decrease in the gap between the mean US US ITE score and mean Rwandan I-ITE score. In addition to overall improvement of the mean scores for all Rwandan residents, we find that the mean Rwandan PG1 I-TE results has increased from − 2 z-scores to − 0.98 z-score, below the non-Rwandan International PG1 scores. This means that the top performing Rwandan post-graduates are now performing at the level of 16% of the other International post-graduates taking the I-ITE. If the rate of improvement was sustained, equity with these other international countries could be obtained within 5–6 years.

US residency programs have implemented different strategies for improving ITE and board performance by residents, including web-based curriculum [20], email-based board review [21], protected block curriculum [22], structured reading and mandatory remediation [23], and accountability program with incentives and consequences [24]. Most of these interventions are not currently utilised in Rwanda. However, there are several plausible explanations for increased Rwandan I-ITE scores, which we outline below:

### Implementation of a formal paediatric curriculum

Prior to the HRH program, residents were taught primarily by means of week-long clusters of lectures delivered a few times per year in an ad-hoc fashion according to local, and primarily Belgian, lecturer availability. Subsequently, a formal curriculum based on the Global Pediatrics Education Consortium was developed and implemented by the HRH and UR faculty during the 2012–2013 academic year [25]. This curriculum was used to guide the development of rotation objectives and of a formal core didactic program that cycled every 2 years. The addition of weekly didactic sessions has been shown to correlate with improved ITE performance in the domains covered by the didactics [26].

Additionally, other modalities of teaching were implemented both at the hospital and university level and included daily morning reports, daily bedside teaching rounds, simulation sessions, practical skill teaching, morbidity, and mortality meetings, research training, and leadership development workshops.

### Improved level of knowledge among clinicians entering the residency program

The mean score of PG1 residents increased during this period. Though PGs sit the I-ITE late in the academic year, the PG1 score is also a surrogate marker for the level of knowledge of residents entering into the residency program. The PG1’s increased level of knowledge suggests that undergraduate education has improved during the period of this study. Implementation of a structured educational curriculum during clinical clerkship has been shown to improve medical student performance on end-of-clerkship shelf exams [27]. In addition to contributing to post-graduate education, the HRH Faculty have also supported undergraduate teaching by reorganizing the paediatric clerkships to include weekly didactics and teaching of practical skills, In addition, HRH Faculty has been involved in undergraduate curriculum revision, in designing new assessment strategies and in supporting undergraduate research projects.

### Change of the official language in Rwanda

Kinyarwanda, English, French, and Kiswahili are the current official languages of Rwanda. English became an official language in Rwanda in 2008. Until then, Kinyarwanda and French were the primary languages of instruction in primary, secondary, and tertiary education. Most of the residents first encountered English as the language of instruction at the tertiary university level. This is similar in Saudi Arabia where most high schools are instructed in Arabic, but the language of instruction in Saudi medical schools is English. A study of Saudi students enrolled in a premedical preparatory course found a correlation between English examination scores and performance on medical written and oral examinations [28]. Therefore, the improved I-ITE scores could be related to improved English proficiency of the Rwandan residents as they advanced in training. In addition, the ABP noticed that some residents might not have been able to complete the I-ITE in the time given and this could have impacted the performance of the residents taking the I-ITE in 2013. In response to this, the ABP increased the time permitted to complete the I-ITE from three to 4.5 h in 2014. This increased time could have contributed to improved scores for the residents taking the I-ITE after 2013.

### Competency in using multiple-choice style questions

Longitudinal exposure to MCQ testing has been shown to improve ITE performance [29]. The first I-ITE in 2012 was the first time any Rwandan resident had taken an examination on a computer, and the MCQ format was not completely familiar to them. In subsequent years there was a greater proportion of residents within the cohort who were previously exposed to the unique computerised format. Therefore, it is possible that the increased scores were due to improved resident familiarity with the electronic platform and question structure format. In addition, since the beginning of HRH, the residents have had access to MCQ practice resources, allowing them to practice taking questions in the same MCQ format as the I-ITE.

### Collaboration and intervention by the HRH faculty

Prior to the start of the HRH program in 2012, there were15 paediatricians in the Rwandan public sector. During the program, there have been 36 expatriate paediatricians working full time, in-country for periods between of 2–3 months (subspecialists) to multiple years (a 1 year minimum for generalists). HRH faculty are integrated as members of the Department of Pediatrics, to work full time in the training environment, supporting clinical and didactic activities at both the undergraduate and postgraduate level. I-ITE scores increased most significantly for the classes that had more years of exposure to the curriculum and teaching and curriculum developed by the Department of Paediatrics Faculty and their embedded HRH colleagues.

## Implication of our results

### Using the I-ITE

The I-ITE is administered in an online, secure, and proctored manner. The individual questions are of uniformly high quality and are standardised by trained question writers following a standardised curriculum. The entire I-ITE is analysed and modified by skilled psychometric analysts. Therefore, we believe that the I-ITE should be highly attractive to paediatric residencies internationally as few paediatric departments have the infrastructure or the faculty with this breadth of technical skills.

The I-ITE can, therefore, be used to provide consistent and detailed formative feedback to individual residents on their pediatric knowledge throughout their residency. The I-ITE is independent of faculty group, in contrast to programs where local faculty design end-of-year internal exams which determine promotion to the next year of training. This lack of reliance on local design leads to recognition of the I-ITE as a tool to monitor the ability of a residency program in general to meet its training requirements over time.

### International training collaboration

These results are highly suggestive that residency organisation and restructuring, through collaborative efforts between local and international faculty, can significantly improve the knowledge competency of residents. The HRH Program aims not only to create a larger, diversified health workforce capable of providing the people of Rwanda with high quality healthcare, but also, through strengthening of academic institutions to develop capacity within Rwanda to carry forward with their own high-quality programs. This study demonstrates that the HRH Program can have an impact on narrowing the gap between resource rich countries and low-resource countries that are facing a shortage of healthcare workforce (including Faculty). However, other competencies cannot be measured with a standardised computer-based testing mechanism and must still be assessed on site. Most importantly, whether and how the increase in scores ultimately impacts clinical practice and patient care remains to be studied.

### Strengths of the study

This is the first study to report the use the I-ITE to measure performance in a resource-limited setting. The results are based on a standardised assessment and therefore void of local bias.

### Limitations of the I-ITE

There are several limitations to our study. The I-ITE is best taken in the first few months of the academic calendar in order to more closely match scores with US counterparts: this was never accomplished due to organisational and financial constraints. Because of administrative and financial issues, we were unable to implement the I-ITE in the 2014–15 academic year, and therefore one data cycle is absent.

The I-ITE only assesses medical knowledge, which is only one of the competencies assessed in a high-quality residency training program. However, the I-ITE has given us a standardised format within which to measure resident medical knowledge. Our personal experience is that clinical skills, attitudes, communication, etc. have also seen parallel improvements during the course of the HRH program.

It is unclear how improved I-ITE scores will impact patient care or residents in the long run. In the US and Canada, ITE scores are used as a predictor of passing internal medicine speciality and subspecialty board examinations [5, 30–33]. There is no objective way to judge if the higher I-ITE scores translate to improved local examination pass rates as there are no standardised speciality certification examinations in Rwanda.

## Conclusions

We demonstrate that the use of a standardised, well-designed, and independent exam available globally can be a powerful tool to monitor the knowledge acquisition of individual residents throughout training. Moreover, we believe it can reflect the capability of residency programs to provide the requisite training. Rwandan pediatric residents improved their pediatric knowledge scores on the I-ITE over a six-year period, both upon entry to residency and throughout their training, possibly reflecting the significant curricular reorganisation accomplished through the HRH Program.

## Data Availability

The datasets generated and/or analysed during the current study are not publicly available but are available from the corresponding author on reasonable request and consultation with the authorising IRB.

## Abbreviations

ABP: American Board of Pediatrics
CHAI: Clinton Health Access Initiative
HRH: Human Resources for Health
I-ITE: International In-Training Examination
IRB: Institutional review board
ITE: In-Training Examination
LMICs: Low-income and middle-income countries
MCQs: Multiple-choice questions
PG: Postgraduate
STROBE: Strengthening the Reporting of Observational Studies in Epidemiology
UAE: United Arab Emirates
UR: University of Rwanda
US: United States
